# Mechanistically informed predictions of binding modes for carbocation intermediates of a sesquiterpene synthase reaction†

**DOI:** 10.1039/c6sc00635c

**Published:** 2016-03-21

**Authors:** T. E. O'Brien, S. J. Bertolani, D. J. Tantillo, J. B. Siegel

**Affiliations:** a Department of Chemistry, University of California Davis Davis California USA djtantillo@ucdavis.edu jbsiegel@ucdavis.edu; b Department of Biochemistry and Molecular Medicine, University of California Davis Davis California USA; c Genome Center, University of California Davis Davis California USA

## Abstract

Sesquiterpenoids comprise a class of terpenoid natural products with thousands of compounds that are highly diverse in structure, generally containing a polycyclic carbon backbone that is constructed by a sesquiterpene synthase. Decades of experimental and computational studies have demonstrated that these enzymes generate a carbocation in the active site, which undergoes a series of structural rearrangements until a product is formed *via* deprotonation or nucleophile attack. However, for the vast majority of these enzymes the productive binding orientation of the intermediate carbocations has remained unclear. In this work, a method that combines quantum mechanics and computational docking is used to generate an all-atom model of every putative intermediate formed in the context of the enzyme active site for tobacco *epi*-aristolochene synthase (TEAS). This method identifies a single pathway that links the first intermediate to the last, enabling us to propose the first high-resolution model for the reaction intermediates in the active site of TEAS, and providing testable predictions.

## Abstract 2

Predicting the binding mode of carbocations produced in sesquiterpene synthase enzymes is not unlike finding a piece of hay in a haystack. A new method for tackling this problem is described.

## Graphical abstract

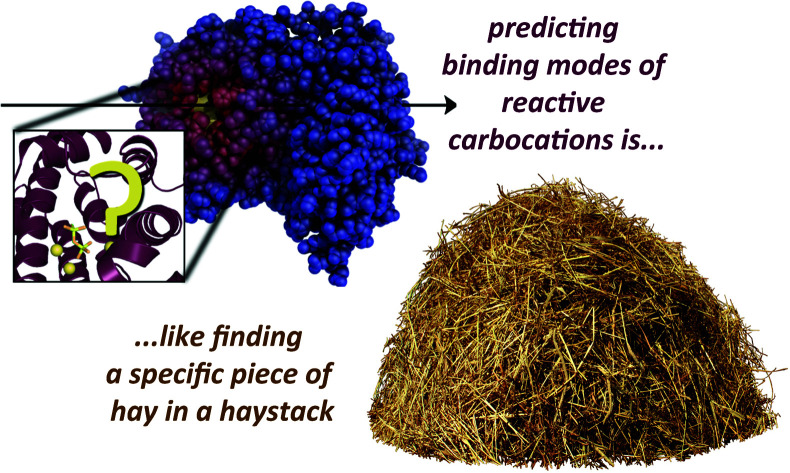

## Introduction

Terpenoids comprise one of the largest families of natural products.^1^ These metabolites play key roles in the realm of chemical ecology^2^ including chemical communication^3,4^—*e.g.*, attracting pollinators,^5^ deterring herbivores^6^—heat protection,^7^ and chemical warfare.^8^ This group of structurally complex metabolites also serves as a diverse chemical library that humans have co-opted for applications in a wide range of areas including agriculture,^9,10^ medicine,^11,12^ and the development of flavors and fragrances.^13^

Terpenes are constructed in nature from simple precursors by a class of enzymes called terpene synthases (or cyclases). These remarkable enzymes generally transform acyclic, achiral substrates into stereodense, polycyclic products. A mechanism by which many of these enzymes work involves magnesium-assisted ionization of an allylic diphosphate to form an allylic carbocation, which undergoes a combination of intramolecular nucleophilic attacks, hydride and alkyl shifts, and proton transfers.^1,14–20^ The enzyme is also thought to play a role in pre-organizing the conformation of the substrate such that once the carbocation has formed there are a limited number of specific products that will be produced.^1^ The results of quantum mechanics (QM) calculations provide support for mechanisms dictated in large part by intrinsic carbocation reactivity.^19,21^

**Fig. 1:**
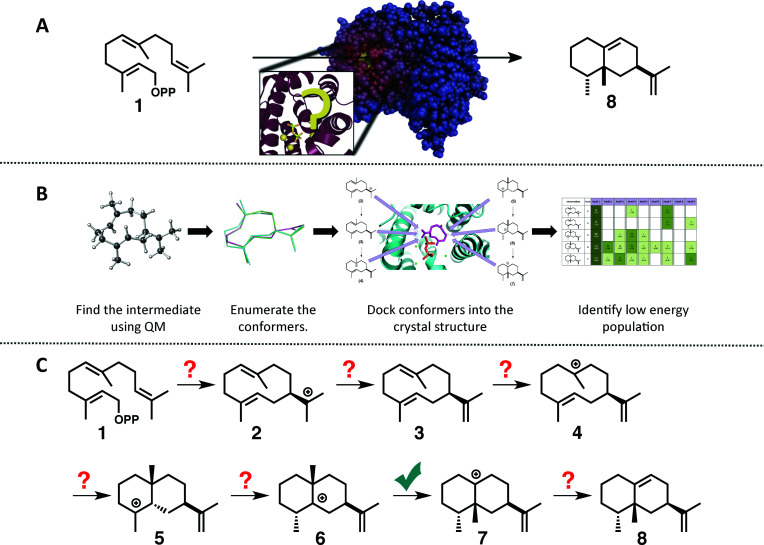
(A) Cartoon emphasizing the difficulty associated with predicting how the substrate orients in the active site of a terpene synthase. (B) Representation of the workflow employed herein: (1) find the intrinsic energy path with QM, (2) enumerate conformers of the intermediates found, but only keep thermally accessible conformers (≤5 kcal mol^−1^ higher than the lowest energy conformer), (3) dock the conformers into the crystal structure using Rosetta and constraints based on known chemistry, (4) filter the data to identify in which orientation the low energy population resides. (C) The generally accepted mechanism for the formation of *epi*-aristolochene (8) in TEAS; the checkmark indicates the only step for which QM calculations have been reported previously.^37^

Although impressive strides have been made in recent years,^22–25^ the ability to build molecular models of entire carbocation cyclization/rearrangement pathways within an active site has remained elusive for the vast majority of terpene synthases (Fig. 1A). As stated by Major and co-workers in a recent review, “A crucial question in any study of terpene synthases is that of the correct binding mode… indeed, crystal structures of terpene synthases often contain substrates bound in unreactive conformations, partly due to the stickiness of the hydrocarbon moiety of the substrate and its lack of hydrogen bond potential. Thus, there is often great uncertainty regarding the correct binding mode when commencing multi-scale simulation projects of terpene cyclases.”^26^ This challenge is likely a major reason why the number of crystal structures available in the protein data bank (PDB) for terpene synthases exceeds 100, while the number of published studies on this family of enzymes that employ QM/molecular mechanics (MM) techniques is less than 10.^19,22,23,26,27^

To tackle this problem we employed a combination of QM calculations to delineate the inherent reactivity of intermediates and computational docking of these intermediates within the enzyme active site. A similar approach was described recently by Jacobson and co-workers in the context of predicting product specificities of terpene synthases;^24,25^ however, our focus is on obtaining mechanistic understanding, guiding future mechanistic experiments and eventually, rational reengineering of this family of enzymes. Here, we employ density functional theory (DFT) calculations rather than semi-empirical calculations,^19^ which will provide a more accurate picture of the potential energy landscape over semi-empirical methods, and we take advantage of the unique features of the Rosetta modelling suite^28–30^ for docking with the incorporation of experimental and mechanism-based constraints (Fig. 1B).^28,30^ In this study we illustrate the utility of this approach for rapidly generating an all-atom model of the reaction pathway for tobacco *epi*-aristolochene synthase (TEAS), a terpene synthase that has been characterized both biochemically and structurally in great detail.^31–34^ In a previous study on *epi*-isozizaene synthase,^35^ we employed an alternative docking approach that lacks many of the features available in Rosetta.^36^ Specifically, Rosetta has the ability to sample significant side chain and backbone conformations, as well as rigid body movements of the ligand. In addition, Rosetta allows the incorporation of user-defined constraints that can encode chemical information about the reaction mechanism that would normally be absent from force-field based molecular modeling approaches.

## Methods

### Quantum mechanics calculations

**Fig. 2:**
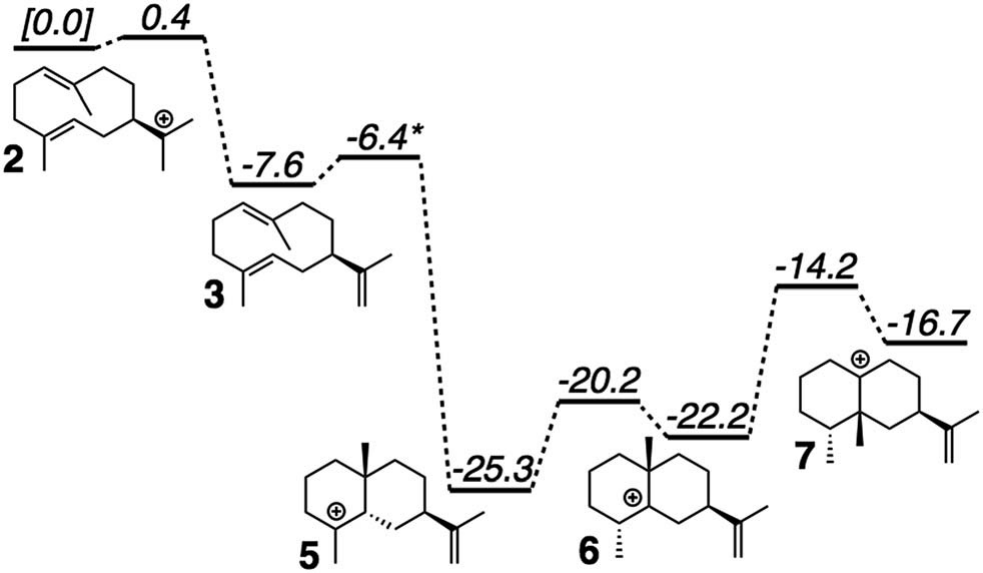
Computed (mPW1PW91/6-31+G(d,p))^40^ relative energies for intermediates and transition state structures involved in the formation of *epi*-aristolochene. *Best estimate based on a variety of computational experiments (see ESI† for details). Relative energies were put onto the same scale by adding in energies of diphosphate and Tyr/Asp theozyme models (not shown; see ESI† for details).

QM calculations were performed with Gaussian09.^46^ Minima and transition state structures (TSSs) were located using mPW1PW91/6-31+G(d,p)^40^ with the SMD continuum solvation approach using dichloromethane, chosen as a crude approximation of the mostly nonpolar active site of the enzyme.^47^ Stationary points were confirmed as minima or TSSs using harmonic vibrational analysis (no imaginary frequencies for minima; a single imaginary frequency for TSSs). Intrinsic reaction coordinate (IRC) calculations^48^ were used to confirm the linkage of a TSS to its flanking intermediates. Fig. 2 shows results for a single set of conformations. All identified intermediates were then subjected to a conformational search using molecular mechanics (MMFF) with Spartan 10.^44^ From these conformational searches greater than one hundred structures per intermediate were identified and then fully optimized using Gaussian09 at the mPW1PW91/6-31G(d) level of theory,^40^ to minimize computational expense while evaluating over 900 conformers total.^19^ All structures found to be within 5 kcal mol^−1^ of the lowest energy conformer were combined to form a library of energetically accessible conformers to be docked into the enzyme.

### Docking calculations

Two crystal structures of TEAS (PDB ID: 5EAT & 4DI5) were minimized using a constrained FastRelax^49^ procedure from the Rosetta modeling suite.^28–30^ The diphosphate/magnesium complex was extracted from another TEAS crystal structure (PDB ID: 3LZ9)^34^, which was docked (unchanged) along with previously generated conformer libraries into both relaxed crystal structures using the chemically meaningful constraints described below (see ESI† for additional details on constraints as well as the 4DI5 docking results). 2500 docking runs per catalytic orientation per intermediate were carried out to ensure that sampling was sufficient. The resulting structures were combined and then filtered by: (1) their ability to meet the constraints – structures that did not satisfy the constraints were not considered, (2) total protein energy – only structures that were one standard deviation or lower from the mean were considered, (3) interface energy – the top 10% in interface energy were selected from the structures that were in the low total protein energy population. These final filtered structures were then grouped by binding orientation to identify where the low energy population resided (Fig. 4).

### RMSD calculations

The entire protein structures were aligned using TMalign^50^ and the RMSD calculation was then performed on each carbon in the skeleton between intermediates, except for the 6 to 7 transition where the shifting methyl group was not considered.

## Results and discussion

### Quantum mechanical modeling of the reaction pathway

The generally accepted mechanism for *epi*-aristolochene formation (Fig. 1C) involves removal of the diphosphate and subsequent nucleophilic attack on the resulting carbocation to form intermediate 2, deprotonation to form germacrene A (3), reprotonation to form 4, cation–alkene cyclization to form 5, 1,2-hydride shift to form 6, 1,2-methyl shift to form 7, and deprotonation to generate the final product, 8. Previous studies on the energetic viability of this pathway have only examined a small portion of the reaction coordinate (Fig. 1C, checkmark).^37^ Therefore DFT calculations (see Methods section for details) were carried out on the pathway shown in Fig. 1C in order to characterize all carbocation intermediates involved in *epi*-aristolochene formation, as well as transition state structures (TSS) connecting them, in terms of both structure and relative energy (in the absence of TEAS). To simulate key portions of the enzyme's active site's effect on the mechanism, two different fragments of the active site were modeled in the QM calculations, termed theozymes. These theozyme calculations have provided insight in the past with defining the role that a key residue might have on the potential energy landscape.^38^ In attempts at finding the 2 to 3 TSS a diphosphate (PPi) was used as the base to deprotonate 2. In attempts to find the 3 to 5 TSS a phenol (to represent tyrosine) and acetate (to represent an activating aspartate) were used.

Minima corresponding to each putative intermediate were found with the exception of carbocation 4 (Fig. 2). Geometry optimizations for intermediate 4 consistently generated intermediate 5, suggesting that protonation and cyclization might be concerted.^19^ Attempts to locate a TSS that directly linked 3 to 5 in the presence of active site groups (see ESI† for details) led only to TSSs for irrelevant processes, likely a result of a flat energy surface near carbocation 3, *i.e.*, the barrier for conversion of 3 to 5 is very small. In order to evaluate this hypothesis, we performed a scan in which the C–H distance (corresponding to protonation of the C=C π-bond) and C–C distance (for the forming C–C bond) were varied while the remainder of the structure was allowed to relax, in the presence of a theozyme^38^ corresponding to the active site Tyr/Asp pair thought to be involved in protonation.^31^ The results of this computational experiment allowed us to estimate that the barrier for conversion of 3 to 5 is likely ≤1 kcal mol^−1^ (see ESI† for details). The remainder of the *epi*-aristolochene pathway did not hold any surprises. Conversion of 5 to 6 and 6 to 7 are predicted to be endothermic steps, however, suggesting that an equilibrium between these species may exist in the active site until site-selective deprotonation (*vide infra*) occurs.^35,39^

### Modeling the reaction pathway in TEAS

We then set out to determine the most probable orientations of the carbocation intermediates in the active site of TEAS, in pursuit of an atomic-resolution model of the entire enzyme promoted pathway. The binding mode of the diphosphate is well-defined in the available crystal structures of TEAS.^41^ Using these crystal structures we performed molecular docking of each intermediate in the reaction pathway within the TEAS active site. During the docking simulations we employed the use of functional constraints based on mechanistic data to enforce molecular interactions that would be required for the enzyme to perform the requisite cyclization chemistry leading to the final product, a key advantage of the Rosetta molecular modeling suite. In these simulations we used three explicit constraints involving: (1) the departing diphosphate oxygen that results in carbocation formation; (2) deprotonation of 2; and (3) protonation of 3.

**Fig. 3:**
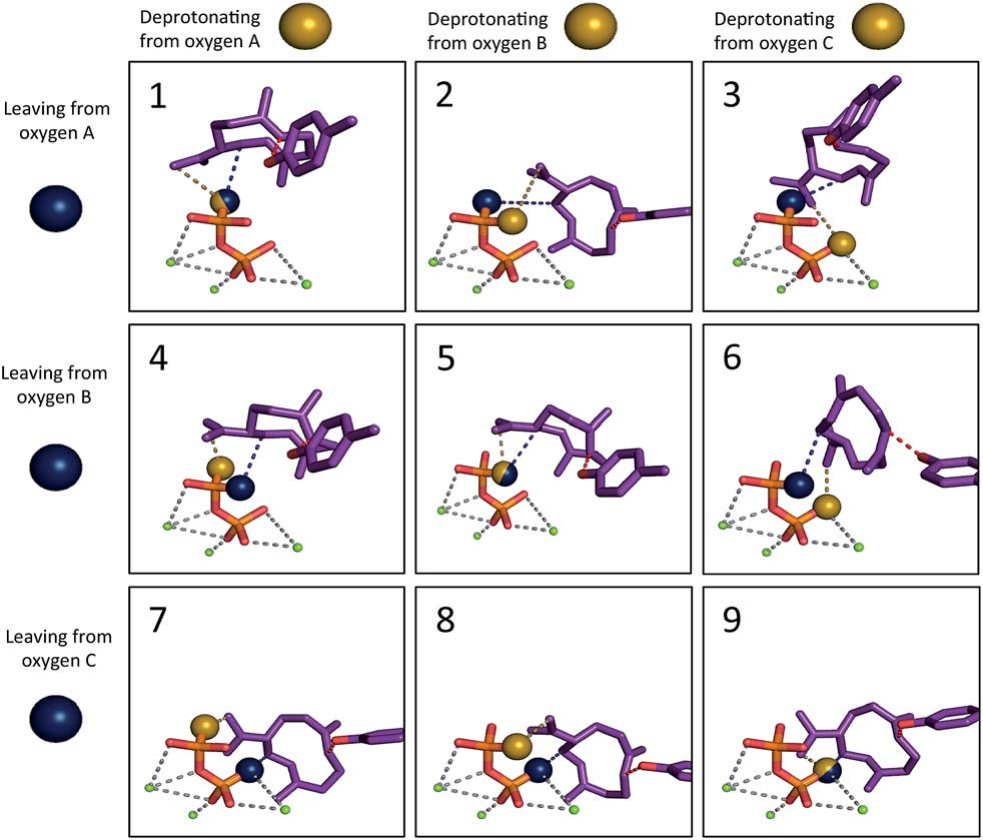
The nine different ion pair orientations (Motifs 1–9). The blue sphere represents the oxygen that was previously bound to the carbon skeleton and was detached to form the carbocation. The yellow sphere represents the oxygen that would play the role of the base in formation of 3. The tyrosine oxygen–alkene carbon constraint is depicted in red. The grey dashed lines illustrate the coordination of magnesium ions (green) to diphosphate.

Based on the TEAS crystal structures, three diphosphate oxygen atoms are pointed into the active site. However, it is not known which of these oxygens was connected to the hydrocarbon portion of the substrate. In addition, it has been hypothesized that one of these three oxygens is the base^18,42^ that deprotonates 2 to form germacrene A (3), but which oxygen is also unknown. Understanding the relative positions of the components of the ion pair is critical to identifying the most likely orientation of the substrate in the pocket. Recent work has demonstrated the importance of careful positioning of the cation and anion in synthetic terpene-forming cyclization/rearrangement reactions.^43^ Given the three possible sites of carbocation disconnection and the three possible sites for deprotonation, nine different possible ion pair orientations arise (Fig. 3, blue and yellow spheres, respectively).

The other mechanism-based constraint was based on the observation reported by Rising *et al.* that mutation of tyrosine 520 to phenylalanine in TEAS changed its activity to that of a germacrene synthase,^31^ implying that this tyrosine may play the role of acid, protonating 3 to give 5. Consequently, the phenolic oxygen of Tyr520 was constrained in our docking calculations to reside near to the carbon of the C=C π-bond that is protonated (Fig. 3, red; see Methods section for details).

Finally, before docking we generated conformational libraries for each of the intermediates along the reaction pathway (Fig. 1B). This was accomplished by subjecting each minimum to a force field-based conformational search followed by a geometry optimization using DFT (at the mPW1PW91/6-31G(d) level of theory) to obtain estimates of each conformation's energy (see Methods section for details).^40,44^ Libraries consisting of conformers within 5 kcal mol^−1^ of the lowest energy conformer for each intermediate were then docked into crystal structures of TEAS using the Rosetta modeling suite.^28–30^

Twenty five hundred independent docking simulations were run for each intermediate conformer library and each set of constraints (*i.e.*, each possible reaction mechanism). This totaled 112 500 independent docking simulations in order to sample the entire reaction pathway space within TEAS. For each intermediate the solution set was filtered based on satisfaction of the constraints, system energy, and interface energy (see Methods for details). All simulations were done in parallel on two different TEAS crystal structures (5EAT and 4DI5) to ensure subtle structural changes did not dramatically change the docking results and, as expected, there were only minor changes between the results of docking simulations when run using different X-ray structures (see ESI†).

**Fig. 4:**
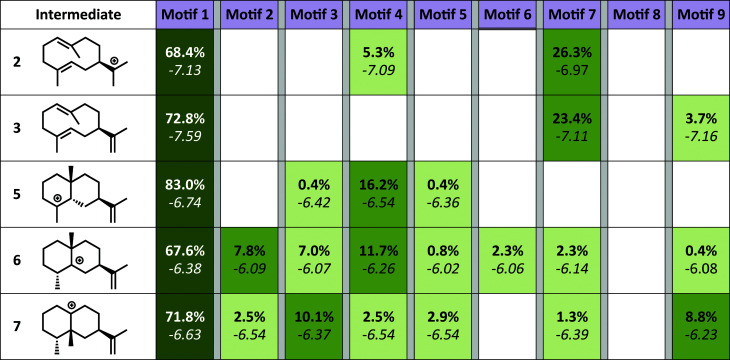
Docking results. Each intermediate is pictured on the left. The darker the green color in each box, the higher the percentage of low energy structures that are found in that catalytic orientation. If no low energy solutions were found for a particular intermediate then no value is given. The number in bold is the percent of total low energy structures found for that that catalytic motif when docking a particular intermediate. The number in italics is the average interface energy (Rosetta energy units) for those structures.

The simulations show a striking enrichment of low energy models for the first reaction mechanism motif with every intermediate (Fig. 4). This result indicates that there is only one binding orientation that links all intermediates to each other – Motif 1. That orientation corresponds to a scenario where the leaving oxygen A (Fig. 3) is also the base that deprotonates intermediate 2. A similar scenario—where the leaving oxygen is also the nucleophile that leads to a diphosphorylated product—was supported by ^18^O-labeling experiments for bornyl diphosphate synthase.^45^ Interestingly, the orientation of substrate analogs in TEAS active sites in crystal structures (PDB codes: 3LZ9, 3M01, 5EAU)^34,41^ would be consistent with Motif 2 (see Fig. F in the ESI†). However, during our simulations very few of the intermediates resulted in low energy conformations in that orientation while satisfying the constraints. This result is consistent with the observation that crystal structures of these enzymes can be misleading in terms of providing insight into the catalytically productive binding mode, especially when conclusions are drawn based on structures with open active sites.^26^

### Structural analysis of reaction pathway from docking

Since a large amount of structural space was sampled during the docking simulations, it is possible that the low energy solutions for any one intermediate are not structurally similar to the low energy solutions for next intermediate. Given how reactive carbocations are (*e.g.*, Fig. 2), we hypothesized that the movement between any two minima is likely to be minimal (*i.e.*, while vibration is reasonable, translation and rotation are less so). Therefore, to obtain an explicit all-atom model of the reaction pathway from beginning to end we identified the low energy structures within Motif 1 with minimal movement between intermediates.

**Fig. 5:**
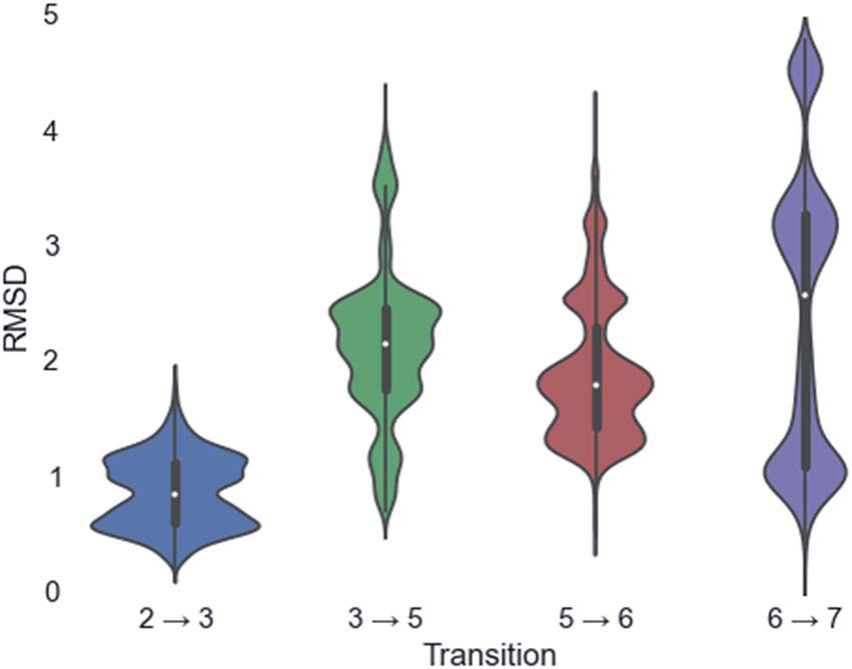
Violin plots of the RMSD calculations for each transition. The white dot represents the mean, the thicker black region represents the first quartile, and the thin black line represents the standard deviation. Mirrored on both sides of the standard deviation line is the population of any given RMSD score.

**Fig. 6:**
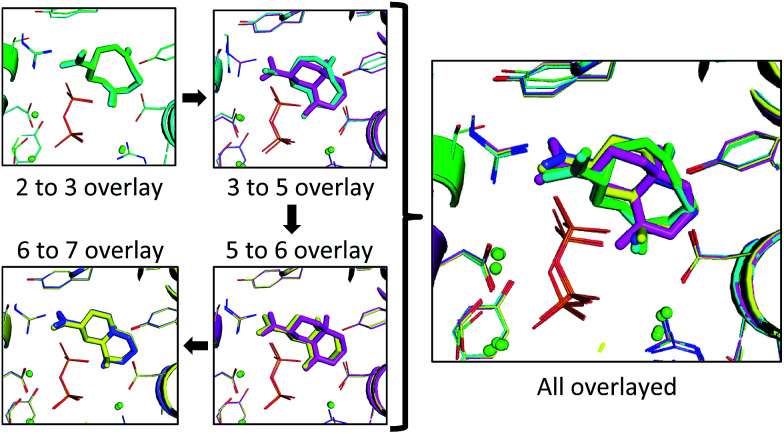
The lowest RMSD structures for each transition (left) and all lowest RMSD intermediates overlayed (right). Each intermediate and the resulting protein structure is represented by a different color: 2 is represented in green, 3 in cyan, 5 in pink, 6 in yellow and 7 in blue. The green spheres correspond to positions of magnesium ions and the diphosphate is shown as sticks in orange (phosphorus) and red (oxygen).

A pair-wise RMSD of carbon atoms was calculated for each pair of connected intermediates for every low energy structure (*e.g.*, carbons in intermediate 2 to 3, 3 to 5, 5 to 6, 6 to 7; Fig. 5). The mean RMSD for the first pair of connected intermediates was 0.88 Å, which indicates that the majority of the structures are in similar structural orientations in the active site. The mean RMSD for each pair increased after the initial 2 to 3 transition. For the 3 to 5 transition the mean RMSD is 2.16 Å, for the 5 to 6 transition it is 1.91 Å and for the 6 to 7 transition it is 2.39 Å. The increased RMSD values for all the transitions after the 2 to 3 transition are due, in part, to a reduction of the ligand volume (see the 3 to 5 overlay in Fig. 6), but are also a result of alternate docking modes.

This is clearest in the 6 to 7 transition, where the average RMSD score appears to have a tri-modal distribution, with one population around 1 Å RMSD, one at 3 Å RMSD and the third at 4.5 Å RMSD. These three populations correspond to three distinct docking orientations (see Fig. G in the ESI†), only one of which aligns well with one of the many docking orientations for intermediate 6. The 2 to 3 transition also appears to have bi-modal character, but in this instance the macrocyclic portion of the structures align very well and the population difference results from two different docking orientations of the exocyclic isopropylene tail in intermediate 3 (see Fig. H in the ESI†), whereas intermediate 2 only has a single docking orientation. If the RMSD is recalculated without taking the tail into account, the average RMSD score for the 2 to 3 transition decreases to 0.68 Å and a more typical Gaussian distribution is observed (see Fig. I in the ESI†).

In the 2 to 3 overlay the overlap is the strongest, with the lowest RMSD for that pair of 0.21 Å, and structures 2 and 3 occupying almost identical spaces (Fig. 6). The 3 to 5 overlay has a higher lowest RMSD value of 0.68 Å, due, in part, to the contraction of volume associated with cyclization (Fig. 6). The lowest RMSD of the 5 to 6 transition is 0.37 Å, which is lower than that for the previous transition. For the 6 to 7 overlay the shifting methyl group was not taken into account for the RMSD calculation, as it would be impossible for it to be in the same position in both structures in that it changes connectivity during the transition. The lowest RMSD for that transition is 0.25 Å. When the lowest RMSD structures for all transitions are overlayed, there is an unambiguous convergence to a single region in the enzyme (Fig. 6, right).

### Predictions to guide future experiments

The binding model presented here leads to (at least) two testable predictions. First, we predict that protonation by Tyr520 would occur on the *re* face of the π-bond; this could be tested by labeling the substrate (*via* chemical synthesis) or the Tyr (*via* the use of D_2_O as solvent). Second, we predict that the diphosphate is not the final base that deprotonates 7 to give 8, since no oxygen in the diphosphate is found to be closer than 5.2 Å from either hydrogen at the position to be deprotonated. We hypothesize that Tyr520 is the final base, with a distance of 2.9 Å in the complex with 7 and an orientation reasonable to remove the pro-*R* hydrogen. This stereochemical prediction could be tested by deuterium labeling of the substrate.

## Conclusions

We present the first all-atom model of the full reaction pathway for TEAS (Fig. 6). Identifying the relative positions of the anionic diphosphate and carbocations in the active sites of terpene synthases is of vital importance for understanding how these enzymes function and provides a basis for further computational studies, *e.g.*, QM/MM dynamics simulations. The method we employ tackles that issue through a combination of QM and computational docking with Rosetta, which allows us to incorporate previous experimental data into the docking, and results in the identification of a single orientation that links all the intermediates along the *epi*-aristolochene-forming pathway. This model allows us to make predictions about the stereochemistry of protonation of 3 and the stereochemistry of deprotonation of 7 (and the identity of the base responsible). Generating such all-atom models of carbocation cyclization/rearrangement pathways in the context of their accompanying protein hosts will enable future efforts to carry out the rational redesign of reaction specificity for this class of enzymes.

## Supplementary Material

SC-007-C6SC00635C-s001

SC-007-C6SC00635C-s002

SC-007-C6SC00635C-s003

SC-007-C6SC00635C-s004

SC-007-C6SC00635C-s005

SC-007-C6SC00635C-s006

SC-007-C6SC00635C-s007

SC-007-C6SC00635C-s008

SC-007-C6SC00635C-s009

SC-007-C6SC00635C-s010

SC-007-C6SC00635C-s011

SC-007-C6SC00635C-s012

SC-007-C6SC00635C-s013

SC-007-C6SC00635C-s014

SC-007-C6SC00635C-s015
