# Supplementary material for: Mechanistically informed predictions of binding modes for carbocation intermediates of a sesquiterpene synthase reaction
Source: Chem Sci. 2016 Mar 21;7(7):4009–15. doi: 10.1039/c6sc00635c (PMC6013805; doi:10.1039/c6sc00635c)
Supplement: SC-007-C6SC00635C-s003 [file SC-007-C6SC00635C-s003.pdf]

Electronic Supplementary Material (ESI) for Chemical Science.
This journal is © The Royal Society of Chemistry 2016

Gaussian Information

Table 1. Below is a table of the energies for the structures reported in the main text of the paper. In addition to the energies all structures are available as mol2 file. Both the sum of the electronic and free energies and electronic energies are listed below, but only the free energies are given in the main text.

**Name**	Int_2	TS_A	Int_3	TS_B	Int_5	TS_C	Int_6	TS_D	Int_7	theozyme	PPi
**Sum of Electronic and Free Energy**											
Abs. Sum En. (Hartrees)	-1797.341	-1797.3403	-1797.3531	-1161.675042	-586.05497	-586.04695	-586.05007	-586.03735	-586.0413	-575.65017	-1211.3263
Abs. Sum En. (kcal/mol)	-1127849.4	-1127849	-1127857	-728962.7058	-367755.36	-367750.32	-367752.28	-367744.3	-367746.78	-361226.24	-760119.35
Abs. Sum En. w/ Theo. (kcal/mol)	-1489075.7	-1489075.2	-1489083.3	Already in calculation	-728981.6	-728976.56	-728978.52	-728970.54	-728973.02		
Abs. Sum En. w/ PPi. (kcal/mol)	Already in calculation	Already in calculation	Already in calculation	-1489082.059	-1127874.7	-1127869.7	-1127871.6	-1127863.6	-1127866.1		
Abs. Sum En. w/ both (kcal/mol)	-1489075.7	-1489075.2	-1489083.3	-1489082.059	-1489101	-1489095.9	-1489097.9	-1489089.9	-1489092.4		
**Rel.** Sum En. To **2** w/ both (kcal/mol)	0	0.4204317	-7.6047937	-6.395745073	-25.28677	-20.25163	-22.211344	-14.226279	-16.706199		
**Rel.** Sum En. To **5** w/ both (kcal/mol)					0	5.03514024	3.07542651	11.0604913	8.58057174		
**HF**											
Abs. Elec. En. (Hartrees)	-1797.7055	-1797.7037	-1797.7211	-	-586.38124	-586.37452	-586.37808	-586.36665	-586.36825	-575.80394	-1211.3527
Abs. Elec. En. (kcal/mol)	-1128078.2	-1128077	-1128088	-	-367960.09	-367955.88	-367958.11	-367950.94	-367951.94	-361322.73	-760135.95
Abs. Elec. En. w/ Theo. (kcal/mol)	-1489400.9	-1489399.8	-1489410.7	-	-729282.82	-729278.61	-729280.84	-729273.67	-729274.67		
Abs. Elec. En. w/ HPPi. (kcal/mol)	Already in calculation	Already in calculation	Already in calculation	-	-1128373.5	-1128369.3	-1128371.5	-1128364.4	-1128365.4		
Abs. Elec. En. w/ both (kcal/mol)	-1489400.9	-1489399.8	-1489410.7	-	-1489418.8	-1489414.6	-1489416.8	-1489409.6	-1489410.6		
**Rel.** Elec. En. To **2** w/ both (kcal/mol)	0	1.15618717	-9.7643694	-	-17.836783	-13.624936	-15.853852	-8.6875622	-9.6885034		
**Rel.** Sum En. To **5** w/ both (kcal/mol)				-	0	4.21184712	1.9829316	9.1492213	8.1482801		
Functional	mpw1pw91	mpw1pw91	mpw1pw91	mpw1pw91	mpw1pw91	mpw1pw91	mpw1pw91	mpw1pw91	mpw1pw91	mpw1pw91	mpw1pw91
Basis Set	6-31+g(d,p)	6-31+g(d,p)	6-31+g(d,p)	6-31+g(d,p)	6-31+g(d,p)	6-31+g(d,p)	6-31+g(d,p)	6-31+g(d,p)	6-31+g(d,p)	6-31+g(d,p)	6-31+g(d,p)
solvent	SMD=DCM	SMD=DCM	SMD=DCM	SMD=DCM	SMD=DCM	SMD=DCM	SMD=DCM	SMD=DCM	SMD=DCM	SMD=DCM	SMD=DCM

Two-dimensional Scan for Identifying the 3 to 5 Transition State Structure.

In our attempt to find a discreet TSS that links 3 to 5 a two-dimensional scan was performed on the carbon-carbon bond forming event, red in **Figure A**, and the carbon-hydrogen bond forming event, blue in **Figure A.** The hydrogen was model as being donated from an activated phenol (a placeholder for tyrosine). The carbon-carbon bond distance was scanned from 1.8 Å to 3.5 Å. The carbon-hydrogen distance was scanned from 1.0 Å to 2.0 Å. The scan was then plotted against energy (**Figure B.**)

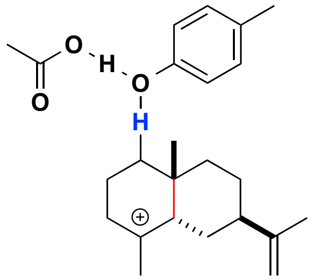

5

Figure A. A chemdraw of the bonds being scanned to understand the potential energy landscape for a TSS, which would like intermediate **3** directly to intermediate **5**.

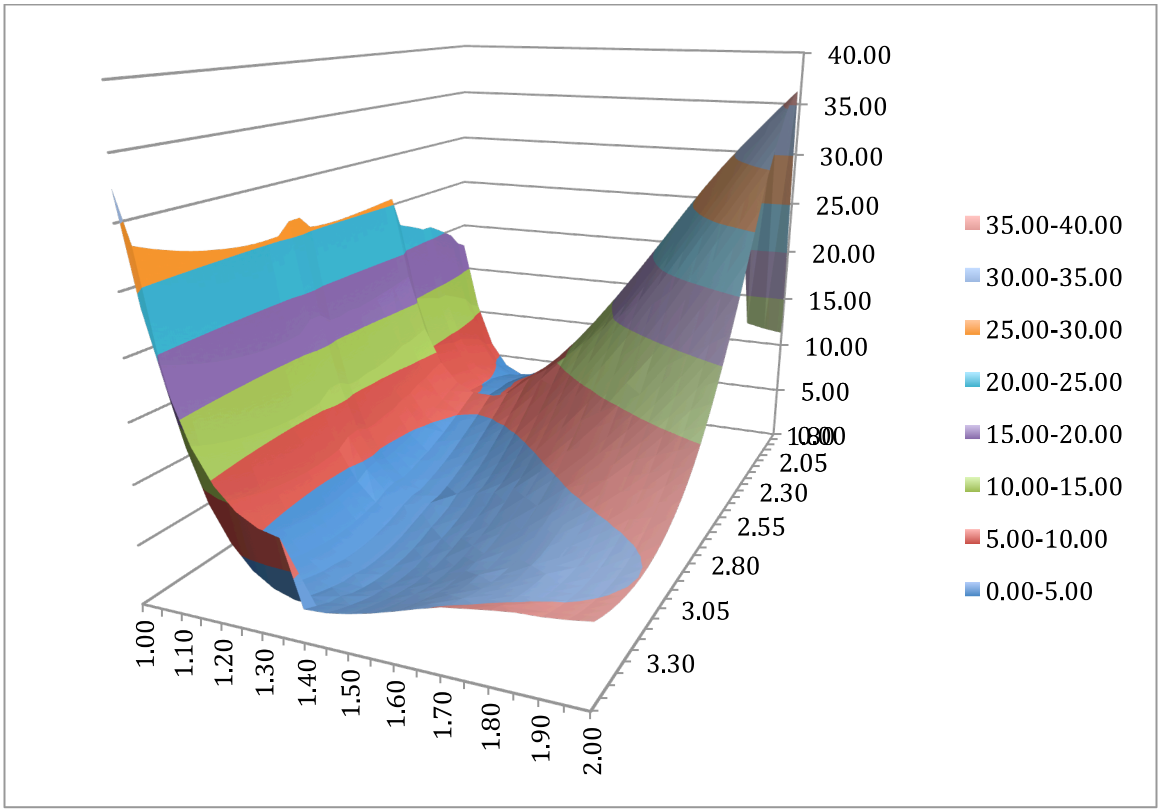

Figure B. The plot of the two dimensional scan. On the side is the legend for the energies (in kcal/mol) that correspond to the colors depicted.

It was hard to tell from **Figure B**, but there was a clear ridge - which may correspond to a TSS. A limited portion of the scan, which better illustrates that ridge is show below as **Figure C**. Multiple points along that ridge were submitted for a TSS, but none came to true TSS.

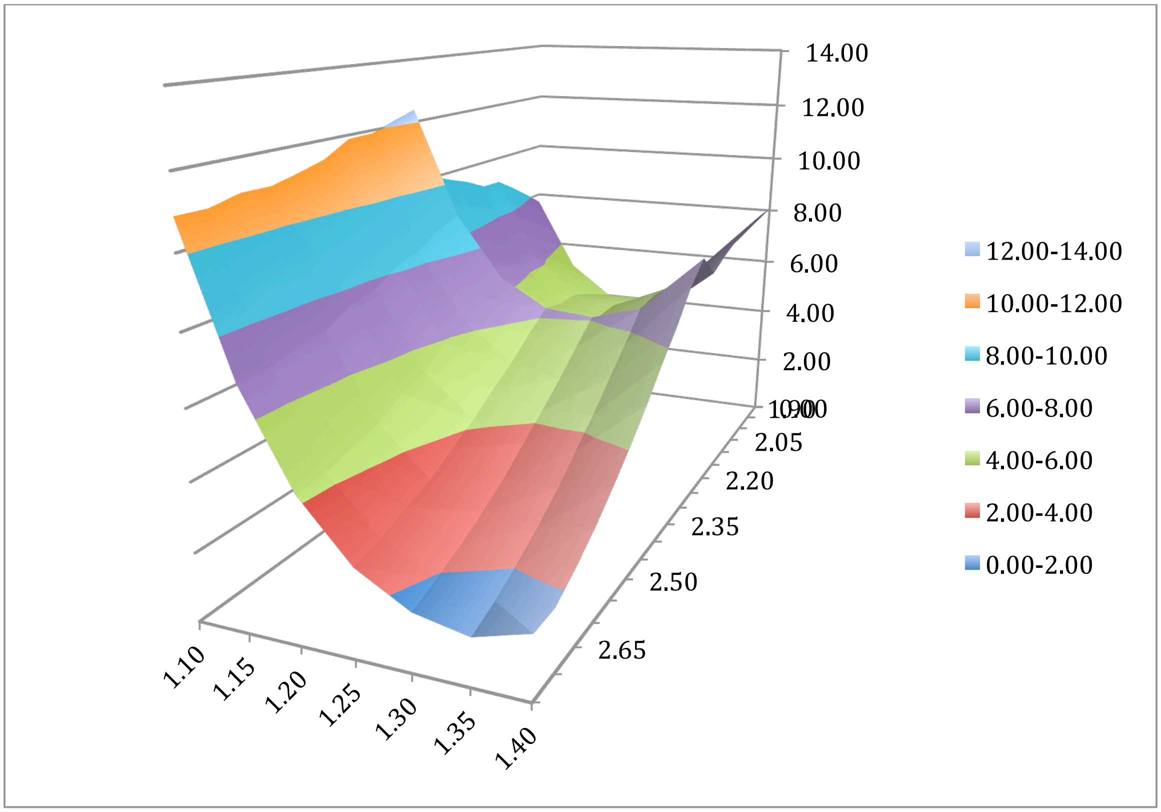

Figure B. Zoom in of the two-dimensional scan above. More clearly illustrated is the ridge thought to be the transition state.

Coordination Constraints

There were nine different constraint files used during the docking – one per each catalytic motif. The majority of the constraints were actually used to dock the Mg/PPi complex into the actives site in an orientation similar to those observed in crystal structures. These are called coordinating constraints, because they are the constraints used to ensure protein coordination to the magnesiums. The coordination constraints generated by measuring the distance, angle and dihedrals from crystal structures that have all three magnesiums and take their average as the constraint value and twice their standard deviation as constraint window (Table 1).

Table 2. Crystal Structures and residues considered for the generation of the coordination constraints. There are also the measured, average and standard deviation values for each of the constraints.

Coordination Constraint	Crystal Structure	Residue number	Oxygen in residue	Distance	Value
4					average distance
Distance	5eat	E452	OE2	2.6	2.60
	3M01	E452	OE2	3.1	standard deviation
	3M00	E452	OE2	2.2	0.45
	3M02	E452	OE2	3	2x STD
	3lz9	E452	OE2	2.1	0.91
					average angle
Angle	5eat	E452	OE2	95.3	103.50
	3M01	E452	OE2	96.5	standard deviation
	3M00	E452	OE2	103	24.28
	3M02	E452	OE2	78.8	2x STD
	3lz9	E452	OE2	143.9	48.57
					average dihedral
Dihedral	5eat	E452	OE2	162.2	157.94
	3M01	E452	OE2	126.7	standard deviation
	3M00	E452	OE2	126.7	37.06
	3M02	E452	OE2	156.8	2x STD
	3lz9	E452	OE2	217.3	74.13

Coordination Constraint	Crystal Structure	Residue number	Oxygen in residue	Distance	Value
5					average distance
Distance	5eat	ASP444	OD1	2.2	2.48
	3m01	ASP444	OD1	2.6	standard deviation
	3m00	ASP444	OD1	2.4	0.18
	3lz9	ASP444	OD1	2.6	2x STD
	3m02	ASP444	OD1	2.6	0.36
					average distance
Angle	5eat	ASP444	OD1	163.2	136.90
	3m01	ASP444	OD1	123.7	standard deviation
	3m00	ASP444	OD1	126.6	21.21
	3lz9	ASP444	OD1	115.2	2x STD
	3m02	ASP444	OD1	155.8	42.43
					average distance
Dihedral	5eat	ASP444	OD1	132.3	150.42
	3m01	ASP444	OD1	158.8	standard deviation
	3m00	ASP444	OD1	166	23.41
	3lz9	ASP444	OD1	175.2	2x STD
	3m02	ASP444	OD1	119.8	46.83

Coordination Constraint	Crystal Structure	Residue number	Oxygen in residue	Distance	Value
6					average distance
Distance	5eat	ASP301	OD1	2.5	2.28
	3m01	ASP301	OD1	2.5	standard deviation
	3m00	ASP301	OD1	2.1	0.23
	3lz9	ASP301	OD1	2	2x STD
	3m02	ASP301	OD1	2.3	0.46
					average distance
Angle	5eat	ASP301	OD1	124.3	136.12
	3m01	ASP301	OD1	128.3	standard deviation
	3m00	ASP301	OD1	138.9	9.50
	3lz9	ASP301	OD1	146.8	2x STD
	3m02	ASP301	OD1	142.3	19.00
					average distance
Dihedral	5eat	ASP301	OD1	109.5	136.90

	3m01	ASP301	OD1	132.9	standard deviation
	3m00	ASP301	OD1	160	20.04
	3lz9	ASP301	OD1	129.4	2x STD
	3m02	ASP301	OD1	152.7	40.08

Coordination Constraint	Crystal Structure	Residue number	Oxygen in residue	Distance	Value
7					average distance
Distance	5eat	ASP301	OD1	2.4	2.24
	3m01	ASP301	OD1	2.1	standard deviation
	3m00	ASP301	OD1	2.1	0.13
	3lz9	ASP301	OD1	2.3	2x STD
	3m02	ASP301	OD1	2.3	0.27
					average distance
Angle	5eat	ASP301	OD1	145.5	131.80
	3m01	ASP301	OD1	97.3	standard deviation
	3m00	ASP301	OD1	135.8	19.74
	3lz9	ASP301	OD1	136.8	2x STD
	3m02	ASP301	OD1	143.6	39.47
					average distance
Dihedral	5eat	ASP301	OD1	155.6	164.72
	3m01	ASP301	OD1	170.7	standard deviation
	3m00	ASP301	OD1	175	8.01
	3lz9	ASP301	OD1	159.3	2x STD
	3m02	ASP301	OD1	163	16.03

Coordination Constraint	Crystal Structure	Residue number	Oxygen in residue	Distance	Value
8					average distance
Distance	5eat	ASP301	OD1	3.7	3.73
	3m01	ASP301	OD1	4	standard deviation
	3lz9	ASP301	OD1	3.6	0.19
	3m02	ASP301	OD1	3.6	2x STD
					0.38
					average distance
Angle	5eat	ASP301	OD1	103.5	99.78
	3m01	ASP301	OD1	92.9	standard deviation
	3lz9	ASP301	OD1	100.3	4.77
	3m02	ASP301	OD1	102.4	2x STD
					9.54

					average distance
Dihedral	5eat	ASP301	OD1	232.3	215.00
	3m01	ASP301	OD1	222.2	standard deviation
	3lz9	ASP301	OD1	195.1	16.00
	3m02	ASP301	OD1	210.4	2x STD
					32.01
3M00 isn't being used for this constraint because the coordination is different than the other crystal structures					

Heatmaps

For a greater explanation on the formation of the heatmap see **Figure D.**

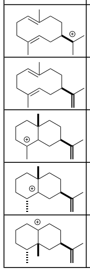

Intermediate	Num	Motif 1	Motif 2	Motif 3	Motif 4	Motif 5	Motif 6	Motif 7	Motif 8	Motif 9
	2	2500	2500	2500	2500	2500	2500	2500	2500	2500
	3	2500	2500	2500	2500	2500	2500	2500	2500	2500
	5	2500	2500	2500	2500	2500	2500	2500	2500	2500
	6	2500	2500	2500	2500	2500	2500	2500	2500	2500
	7	2500	2500	2500	2500	2500	2500	2500	2500	2500

a) Filter based on constraint energy

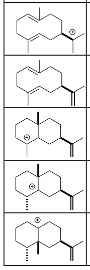

Intermediate	Num	Motif 1	Motif 2	Motif 3	Motif 4	Motif 5	Motif 6	Motif 7	Motif 8	Motif 9
	2	634	823	566	808	746	915	838	277	643
	3	833	850	564	461	724	346	750	368	875
	5	2050	1207	2082	1905	1640	1856	1891	1068	1759
	6	1884	1720	1918	1992	1978	2060	1808	1125	1798
	7	2005	2095	2036	2098	1889	2077	1796	1523	1933

b) Filtered based on total protein energy

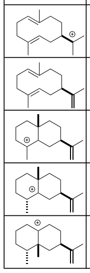

Intermediate	Num	Motif 1	Motif 2	Motif 3	Motif 4	Motif 5	Motif 6	Motif 7	Motif 8	Motif 9
	2	287	44	147	108	3	12	285	17	48
	3	322	52	92	32	5	1	111	9	174
	5	931	16	425	272	7	97	376	8	238
	6	780	82	641	141	18	179	281	26	403
	7	750	163	602	110	36	136	205	45	398

c) Filter based on interface energy.

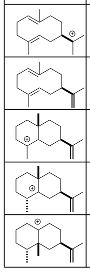

Intermediate	Num	Motif 1	Motif 2	Motif 3	Motif 4	Motif 5	Motif 6	Motif 7	Motif 8	Motif 9
	2	65			5			25		
	3	59						19		3
	5	200		1	39	1				
	6	173	20	18	30	2	6	6		1
	7	171	6	24	6	7		3		21

Figure D. Workflow of the energy based filtering method utilized to identify the putative low energy reaction pathway. A total of 2500 docking simulations was conducted with each intermediate for each of the nine putative reaction motifs. The numbers in each box in the figure is a count of the remaining structures after each filtration step. (a) Of the resulting 112,500 docked structures any that did not have an intermediate bound in a geometric orientation that satisfied the motif constraints (defined as a constraint energy of > 1 Rosetta energy unit) were removed. (b) Since both the protein side chain and backbone were allowed to move during the simulations a second filter to identify low energy protein structures was applied. All motifs for each intermediate were pooled and structures ≥1 standard deviation below the mean total system energy were kept. (c) In order to identify putative low energy binding modes a third and final filter was applied to the remaining structures based on the calculated interface energy. All motifs for each intermediate were pooled and the lowest 10% in interface energy were kept.

In the main text of the paper only the percentage of low energy structure were shown. Included below are the absolute numbers of structures found for each catalytic orientation or motif for docking into the 5EAT crystal structure.

Intermediate	Num	Motif 1	Motif 2	Motif 3	Motif 4	Motif 5	Motif 6	Motif 7	Motif 8	Motif 9
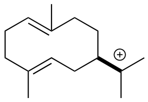	2	**65**<br>*-7.13*			**5**<br>*-7.09*			**25**<br>-6.97		
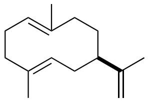	3	**59**<br>*-7.59*						**19**<br>*-7.11*		**3**<br>*-7.16*
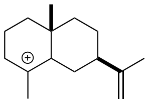	5	**200**<br>*-6.74*		**1**<br>*-6.42*	**39**<br>*-6.54*	**1**<br>*-6.36*				
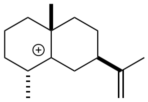	6	**173**<br>*-6.38*	**20**<br>*-6.09*	**18**<br>*-6.07*	**30**<br>*-6.26*	**2**<br>*-6.02*	**6**<br>*-6.06*	**6**<br>*-6.14*		**1**<br>-6.08
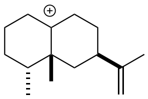	7	**171**<br>*-6.63*	6<br>*-6.54*	**24**<br>*-6.37*	**6**<br>*-6.54*	**7**<br>*-6.54*		**3**<br>*-6.39*		**21**<br>*-6.23*

Figure E. Heatmap for docking in the 5EAT crystal structure with the absolute numbers of structures per orientation instead of the percentages found per orientation.

In addition to docking the intermediates in the 5EAT crystal structure, the intermediates were also docked into an alternate TEAS crystal structure to ensure the results were not because of input bias. Below is the heatmap for docking into this alternate crystal structure – 4DI5. This crystal structure also identifies the motif 1 as the most likely catalytic orientation. This structure does have an alternate motif that links the first intermediate to the last, but many of those are a very low number or solutions. Specifically, intermediate **5** is only 1% of the low energy structures and intermediate **7** is less than 1%. These small percentage of solutions are within the inherent noise of the monte-carlo simulations conducted with the Rosetta modeling suite and thus aren't likely to be predictive.

Intermediate	Num	Motif 1	Motif 2	Motif 3	Motif 4	Motif 5	Motif 6	Motif 7	Motif 8	Motif 9
Leaving - Deprot		A - A	A - B	A - C	B - A	B - B	B - C	C - A	C - B	C - C
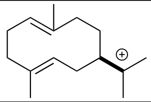	2	**45**<br>*-7.30*	**20**<br>*-5.93*	**1**<br>*-5.93*	**1**<br>*-6.09*	**1**<br>*-5.77*	**1**<br>*-5.98*	**44**<br>*-6.30*	**1**<br>*-5.95*	**7**<br>*-5.80*
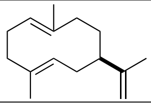	3	**26**<br>*-8.23*						**63**<br>*-7.42*		**6**<br>*-7.68*
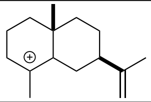	5	**222**<br>*-7.54*			**20**<br>*-7.09*					**2**<br>*-6.90*
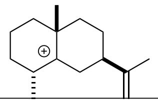	6	**371**<br>*-6.58*	**99**<br>*-6.67*	**15**<br>*-6.17*	**294**<br>*-6.43*	**25**<br>*-6.17*	**2**<br>*-6.15*	**7**<br>*-6.34*	**3**<br>*-6.15*	**26**<br>*-6.23*
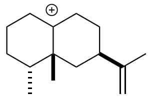	7	**154**<br>*-7.48*	**56**<br>*-7.29*	**11**<br>*-7.25*	**13**<br>*-7.57*	**8**<br>*-7.62*	**3**<br>*-7.57*			**2**<br>*-7.17*

Figure F. Heatmap for docking into alternate crystal structure of epi-aristolochene synthanse PDB code 4DI5.

Misleading Crystal Structures.

Based on the orientation in crystal structures one would expect that motif 2 would be the most likely to score well (**Figure G1**). There are three different crystal stuctures that have similar binding orientations (**Figure G2**). Based on this methodology, that orientation scores poorly.

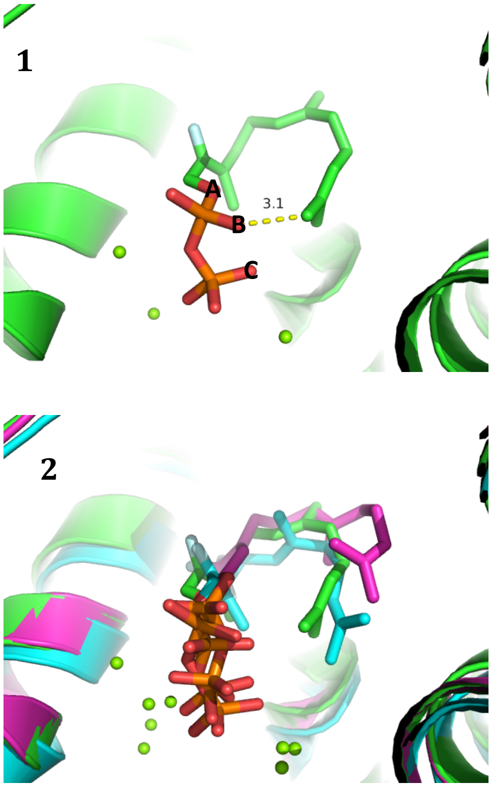

Figure G. Crystal structures that would predict motif 2 as the most likely. (**1**) Crystal structure 3LZ9 with the three oxygens labeled according the constraints chart (**Figure 3**) in the main text. The distance labeled is the distance to the carbon to deprotonate from oxygen B. (**2**) The three crystal structures cited in the main text that all appear to support orientation 2 as the most likely. The green strucute is 3LZ9, the cyan structure is 3M01 and the magenta structure is 5EAU. The substrate analog in 3LZ9 and 3M01 is 2-fluorofarnesyl diphosphate. The substrate in 5EAU is 3-trifluromethylfarnesyl diphosphate.

The Trimodal Distribution.

Included here are representative structures for the populations that form up the trimodal distribution found in the **6** to **7** RMSD calculation (**Figure H**).

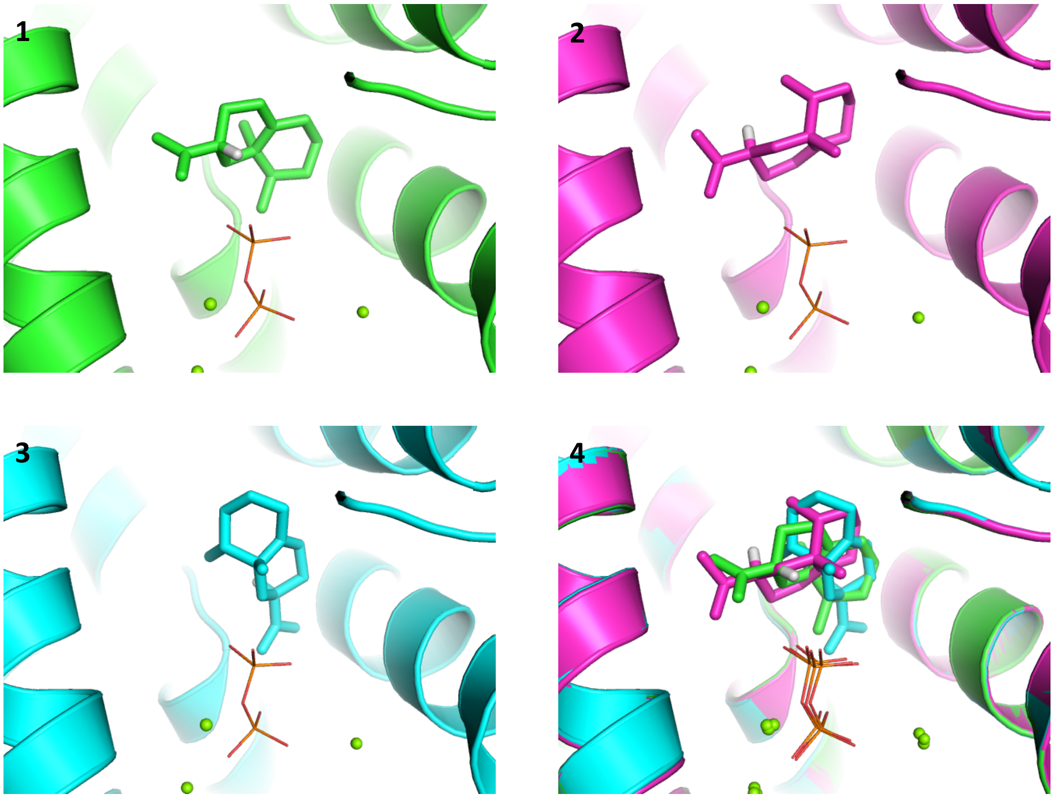

Figure H. The three binding modes found in the trimodal distribution in the RMSD calculation. (**1**) This is the low RMSD population, which aligns well with structures from intermediate 6 (**2**) This is the docking orientation with a RMSD values ~ 3Å. This population is 180° rotation from the orientation in 1. (**3**) This is representative of the highest RMSD docking orientation. (**4**) This is all three docking orientations aligned together.

Bimodal Distribution

In the main text the bimodal appearance of the **2** to **3** transition is largely a result of the conformational freedom in the isopropylene tail in intermediate **3** (**Figure I**). When the RMSD is recalculated for that transition, the shape of the distribution goes to a more gaussian-like distribution (**Figure J**).

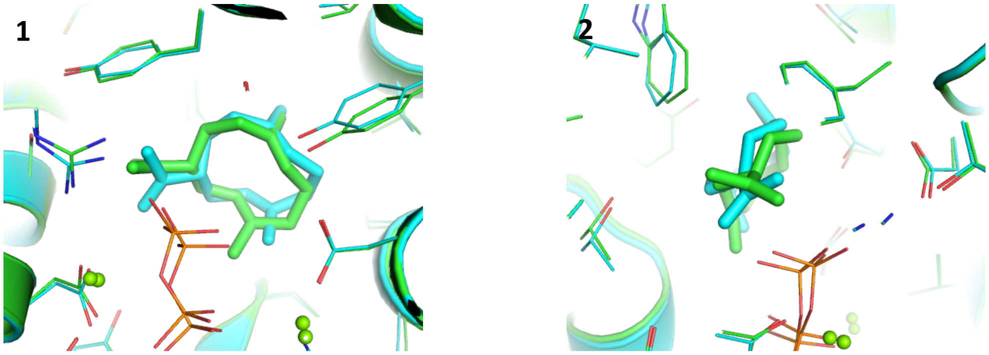

Figure I. The overlays of the **2** to **3** transition which show: (**1**) that the macrocycle aligns wells and (**2**) that the bimodal appearance of the all atom RMSD (**Figure 5** – main text) was largely a result of the two different orientations of the tail.

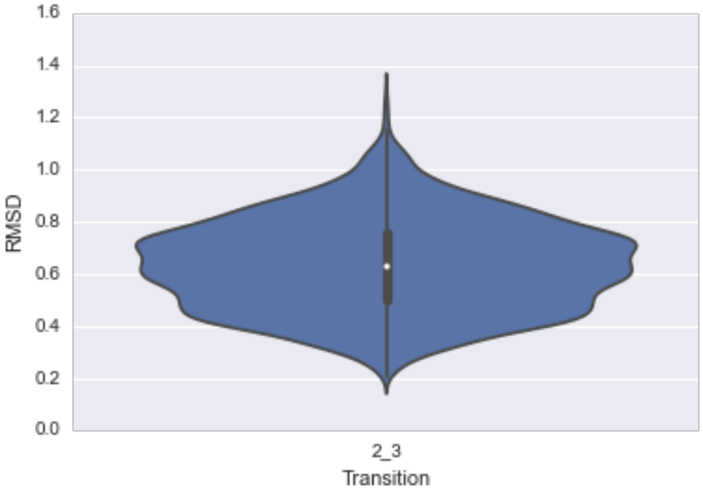

Figure J. Violin plot of the **2** to **3** transition calculated without the isopropylene tail. The distribution without the tail is gaussian.

Partial charges of cations in Rosetta

Rosetta doesn't have discrete terms for the handling of carbocations in its scoring function. Rosetta does assign partial charges to all atoms, but those partial charges, which are derived from QM calculations, are design for handling proteins. To investigate the impact of the partial charges on the results, the partial charges that *Rosetta* adds were overwritten with two different partial charges from QM calculations. The first partial charges were taken from the Mulliken charges, the second set of charges were taken from the calculated electrostatic charges from the flag pop=chelpg option in *Gaussian09*. Intermediate **7** was docked with these three different partial charge options (**Figure K**). It is clear that the type of partial charge does not affect the results of the docking, where all three options identify the same catalytic motif as the most likely. This result led us to use the default charges for Rosetta as a way to make the method as generalizable as possible. That the partial charges don't make a bigger difference leads us to conclude docking in our simulations is dominated by shape. In addition, many of the physical properties that chemists attribute to electrostatics are included in other portions of the scoring function – e.g., hydrogen bonding is covered by its own energy term. As of yet, *Rosetta* doesn't include terms for non-classical hydrogen bonding interactions, such as cation-π interactions; that is something that our group is working on adding.

1

Motiff	number of solutions	normalized	Avg Int E	Low Int E
m1	171	69.5	-6.63	-7.84
m2	14	5.7	-6.32	-6.73
m3	24	9.8	-6.37	-6.74
m4	6	2.4	-6.54	-7.20
m5	7	2.8	-6.54	-7.44
m6	0	0.0	-	-
m7	3	1.2	-6.39	-6.54
m8	0	0.0	-	-
m9	21	8.5	-6.23	-6.82

2

Motiff	number of solutions	normalized	Avg Int E	Low Int E
m1	150	58.6	-6.91	-8.18
m2	12	4.7	-6.67	-7.19
m3	28	10.9	-6.52	-7.08
m4	14	5.5	-6.70	-7.48
m5	7	2.7	-6.67	-7.20
m6	0	0.0	-	-
m7	5	2.0	-6.56	-6.99
m8	1	0.4	-6.40	-6.40
m9	39	15.2	-6.54	-7.24

3

Motiff	number of solutions	normalized	Avg Int E	Low Int E
m1	156	74.3	-6.62	-7.91
m2	13	6.2	-6.26	-6.54
m3	11	5.2	-6.38	-6.72
m4	6	2.9	-6.42	-6.66
m5	6	2.9	-6.42	-6.84
m6	0	0.0	-	-
m7	0	0.0	-	-
m8	0	0.0	-	-
m9	18	8.6	-6.18	-6.52

Figure K. Docking results for the three different partial charges on intermediate **7**. *1)* (*grey header)* The default partial charges applied by Rosetta and reported in the main text of the paper. 2) *(red header)* The partial charges were manually changed to the muliken charges calculated for intermediate **7** in the QM. *3) (blue header)* Docking results when the calculated electrostatic potential charge replaced the default charges used in rosetta. Although specific numbers change slightly there wasn't significant difference in the results regardless of which partial charges were used.
